# Production of Lipopeptide Biosurfactant by a Marine *Nesterenkonia* sp. and Its Application in Food Industry

**DOI:** 10.3389/fmicb.2017.01138

**Published:** 2017-06-28

**Authors:** George S. Kiran, Sethu Priyadharsini, Arya Sajayan, Gopal B. Priyadharsini, Navya Poulose, Joseph Selvin

**Affiliations:** ^1^Department of Food Science and Technology, Pondicherry UniversityPuducherry, India; ^2^Department of Microbiology, School of Life Sciences, Pondicherry UniversityPuducherry, India

**Keywords:** marine actinomycetes, lipopeptides, biosurfactant, antibiofilm, bioemulsifier

## Abstract

Biosurfactants are smart biomolecules which have wide spread application in medicines, processed foods, cosmetics as well as in bioremediation. In food industry, biosurfactants are used as emulsion stabilizing agents, antiadhesives, and antimicrobial/antibiofilm agents. Nowadays biosurfactant demands in industries has increased tremendously and therefore new bacterial strains are being explored for large scale production of biosurfactants. In this study, an actinobacterial strain MSA31 was isolated from a marine sponge *Fasciospongia cavernosa* which showed high activity in biosurfactant screening assays such as drop collapsing, oil displacement, lipase and emulsification. Lipopeptide produced by MSA31 was found to be thermostable which was evident in differential scanning calorimetry analysis. The spectral data obtained in the Fourier transform infrared spectroscopy showed the presence of aliphatic groups combined with peptide moiety which is a characteristic feature of lipopeptides. The stability index of lipopeptide MSA31 revealed “halo-alkali and thermal tolerant biosurfactant” which can be used in the food industry. Microtiter plate assay showed 125 μg/ml of lipopeptide was effective in reducing the biofilm formation activity of pathogenic multidrug resistant *Staphylococcus aureus*. The confocal laser scanning microscopic images provided further evidences that lipopeptide MSA31 was an effective antibiofilm agent. The antioxidant activity of lipopeptide MSA31 may be due to the presence of unsaturated fatty acid present in the molecule. The brine shrimp cytotoxicity assay showed lipopeptide MSA31 was non-toxic and can be used as food additives. Incorporation of lipopeptide MSA31 in muffin showed improved organoleptic qualities compared to positive and negative control. This study provides a valuable input for this lipopeptide to be used in food industry as an effective emulsifier, with good antioxidant activity and as a protective agent against *S. aureus*.

## Introduction

Marine sponges are the reservoir of dense and unique marine microbial communities. The sponge tissue is a microbial niche which provides favorable conditions for the abundant growth of marine microorganisms. About 40% of sponge biomass comprises of complex microbial communities (Selvin et al., 2010). Actinomycetes are most abundant bacterial genera comprising about 20% of microbiome in some marine sponges (Montalvo et al., 2005; Gandhimathi et al., 2008). The metabolites derived from sponge associated actinomycetes show a wide range of biological activities which include antibacterial, antifungal, antiparasitic, antimalarial, immunomodulatory, anti-inflammatory, antioxidant, and anticancer properties (Selvin and Lipton, 2004; Bull and Stach, 2007; Pimentel-Elardo et al., 2010; Abdelmohsen et al., 2012; Blunt et al., 2013). Sponge associated actinomycetes were first reported biosurfactant producers among the group “actinomycetales" (Gandhimathi et al., 2009). Biosurfactants and bioemulsifier production has been increased in recent years as they were important green compounds which showed application in medicines, processed foods, cosmetics, and environmental cleaning (Kiran et al., 2010b).

Biosurfactants are compounds which have high surface tension reducing property along with emulsification activity whereas bioemulsifiers possess high emulsification activity with less or negligible surface tension reducing property. Biosurfactant and bioemulsifier production has increased in recent years as these biomolecules derived from actinomycetes showed tremendous application in medicine, processed foods, cosmetics and in bioremediation (Kiran et al., 2010b). Biosurfactants have multifunctional applications in food industry as emulsifiers, antiadhesives, and antimicrobial/antibiofilm agents. Biosurfactants are emulsion stabilizing agents, incorporated in food products to maintain the consistency, texture, solubilisation of fat globules, improve aroma, foaming and dispersing properties (Campos et al., 2013). Bioemulsifier is a natural food ingredient which can be used to improve the rheology of dough, increase the volume and emulsification of fat thus finds further applications in bakery and meat processing industry (Kourkoutas et al., 2004).

Considering the increased use of bioemulsifier in food industry, identification of new compounds with low or no toxicity and high emulsifying property has become essential Chemically synthesized surfactants are toxic whereas biosurfactants obtained from microbes are non-toxic and/or with least toxicity and they are highly stable at extreme temperature, pH and salinity. In food, rhamnolipid biosurfactant was used to improve the quality of the baked and confectionary products (Van Haesendonck and Vanzeveren, 2004). Incorporation of 0.10% rhamnolipid has been found to improve the texture and shelf life of muffin and croissants (Gandhi and Skebba, 2007). Bioemulsifier produced from marine *Enterobacter cloacae* has been used to enhance the viscosity of acidic food products (Shepherd et al., 1995; Iyer et al., 2006). Even though biosurfactants as bioemulsifiers have immense application in food industry, reports on their utilization in food industries are very limited.

In this study, a marine sponge associated actinomycetes *Nesterenkonia* sp. MSA31 was screened for biosurfactant production and was chemically characterized as lipopeptide derivative. The lipopeptide MSA31 showed no toxicity in brine shrimp cytotoxicity assay and acted as an antibiofilm agent against multi drug resistant *Staphylococcus aureus.* In this report, we demonstrate that incorporation of lipopeptide bioemulsifier in muffin preparation shows improved softness and its organoleptic quality.

## Materials and Methods

### Isolation and Screening of Actinomycetes

The actinomycetes used in this study for biosurfactant production was isolated from a marine sponge *Fasciospongia cavernosa* collected by SCUBA diving at a depth of 10–15 m in Vizhinjam 8°22′45″N 76°59′29″E located in southwest coast of India. The sponge samples were immediately transported to the laboratory in ice box. Approximately 1 cm^3^ of the sponge tissue was excised using sterile scissors and washed extensively in sterile seawater. The sponge tissue was homogenized using a tissue homogenizer. The aliquot was serially diluted in sterile seawater and plated on various media as mentioned in our previous report (Gandhimathi et al., 2009). The isolates obtained were cultured on a minimal salt media containing g/L of KH_2_PO4 0.5 g, FeSO_4_ 7H_2_O 0.1 g, Na_2_CO_3_ 0.2 g, L-asparagine 0.1 g, MgSO_4_ 0.1 g, yeast extract 1 g and NaCl 1 g, pH 7. After 144 h of incubation, the culture media were centrifuged at 14,000 rpm for 20 min (Eppendorf). The filtrate obtained was screened on various screening assays including drop collapsing (Jain et al., 1991), oil displacement (Morikawa et al., 2000), lipase (Kiran et al., 2010b), haemolytic activity (Kiran et al., 2010b), surface tension measurement (dynamic tensiometer), and emulsification index (Paraszkiewicz et al., 1992). The emulsification index was determined using cell free supernatant (CFS) and sunflower oil (Goldwinner^®^) as substrate (1:1), the mixture was vortexed for 60 s and stability of the formed emulsion was determined after 24 h.

### Identification of Biosurfactant Producer

The genomic DNA of MSA31 was isolated by CTAB/NaCl method. Universal 16S rRNA eubacterial primer (5′-GAGTTTG ATCCTGGCTCAG-3′; 5′-AGAAAGGAGGTGATCCAGCC-3′) was used for the amplification of DNA. The 16S rRNA gene sequence obtained from the producers was compared with other bacterial sequences by using NCBI BLASTn (Altschul et al., 1990, 1997) for their pair wise identities. Multiple alignments of these sequences were carried out by Clustal W 1.83 version of EBI^1^ with 0.5 transition weight. Phylogenetic trees were constructed in MEGA 7 version^2^ using Maximum Parsimony (MP) method.

### Extraction and Purification of Biosurfactant

The isolate MSA31 was inoculated into 1 L of production media composed of minimal salt media enriched with 10 g olive oil, 10 g ammonium nitrate and 19 g NaCl and incubated at 28°C for 144 h with agitation of 180 rpm. After incubation, the CFS was obtained by centrifugation at 14,000 rpm for 20 min at 4°C (Eppendorf). The supernatant was acidified to pH 2.0 with 0.1N HCl and allowed to form precipitate by incubating overnight at 4°C. The acid precipitate was collected by centrifugation at 12,000 rpm for 30 min, 4°C. The precipitated biosurfactant was washed several times with sterile distilled water and the pH was adjusted to 7.0 using 0.1 N NaOH. The precipitate was resuspended in sterile distilled water and solvent optimization was performed by adding equal volume of extraction solvents such as methanol, ethyl acetate, diethyl ether and dichloromethane (v/v). The resultant aliquot was concentrated to dryness in a rotary vacuum evaporator (Yamato). The solvent extract with high emulsification activity was further purified using column chromatography on silica gel (60–120 mesh) with step wise elution with methanol and water ranging from 65 to 100% (v/v) at a flow rate of 0.5 ml/min at room temperature (27°C). The purified fraction was used for chemical characterization and identification of active molecule.

### Chemical Characterization of Biosurfactant Compound

The active fraction was confirmed by the emulsification activity and the purity was checked by TLC. The TLC fractionation was performed using the solvent system for the separation of biomolecules which include proteins (*n*-butanol: acetic acid: water 45:35:20), carbohydrates (chloroform: acetic acid: water 50:30:20) and lipids (chloroform: methanol: water 60:30:10). The spots developed on the TLC plates were visualized by spraying of 50% H_2_SO_4_ for carbohydrates and ninhydrin for amino acids. The TLC plates were exposed in an iodine chamber to visualize the lipid fractions (Kiran et al., 2010b). To determine the functional groups the purified active column fraction was lyophilised in a lyophilizer (Yamato DC 400) and subjected to FT-IR analysis. The lyophilised active fraction was used for FTIR analysis on a Bruker IFS113v FTIR spectrometer, in the 4000–400 cm^-1^ spectral region at a resolution of 2 cm^-1^ and 50 scans. The lyophilized active fraction was investigated by using Gas chromatography (GC) (Perkin Elmer Autosystem XL GC-model Clarus-680, United States). For the structure prediction one dimensional ^1^H NMR spectra were recorded by dissolving the biosurfactant at a concentration of 25 mg/ml^-1^ in deuterated DMSO and analyzed on a Bruker AVANCE III 500 MHz. C NMR was recorded on a solid state NMR (400 MHZ – JEOL-ECX-400).

### Stability of Biosurfactant

The column purified fraction of biosurfactant was evaluated for its stability at different temperature, pH and salt concentrations. The stability assays were performed as per Kiran et al. (2010b). To determine the pH stability, the biosurfactant 0.4% (w/v) was dissolved in various pH ranges of buffer solutions which include 0.1 M sodium acetate buffer (pH4.0–7.0) and 0.1 M sodium phosphate buffer (pH 8.0–9.0). The aliquot was incubated at 37°C for 1 h to determine pH stability based on emulsification index. Similarly, the biosurfactant 0.4% (w/v) was dissolved in distilled water containing various concentrations of NaCl ranging between 1 and 12% and incubated at 37°C for 1 h to determine NaCl stability. Temperature stability was determined by incubating the biosurfactant at various temperatures ranging between 4 and 121°C for 1 h, and then the stability of the compound was assessed based on the emulsification index.

### Thermal Gravimetric (TG) and Differential Scanning Calorimetric (DSC) Analysis

Thermal Gravimetric and Differential Scanning Calorimetric of biosurfactant were performed and determined using TA instruments, Q600 SDT and Q20 DSC (Kiran et al., 2014). Approximately 3 mg of sample was placed in an aluminum pan. The analysis was carried out over the temperature range from 0°C to 300°C at a rise in temperature of 10°C/min. The flow rate of the gas was set at 50 ml/min.

### Antioxidant Activity

Antioxidant activity of the biosurfactant from MSA31 was analyzed using 2,2-diphenyl-1-picryl hydrazyl (DPPH) radical scavenging assay (Turkmen et al., 2006) with necessary modifications. In this assay, 0.3 ml containing different concentrations of biosurfactant between 0.5 and 6 mg/ml were added to 3.5 ml of 99.5% ethanol containing DPPH (0.02 mM). The assay mixture was mixed well and incubated in dark at 25°C for 30 min. Butylated hydroxytoluene (BHT) was used as the positive control, ethanol was set as blank and the assays were performed in triplicates. The process of decolourization was recorded at 520 nm using Shimadzu UV-VIS spectrophotometer. The percentage of radical scavenging was calculated using the following formula.

$$AA(\%)=[({Abs.}_{control}-{Abs.}_{sample})/{Abs.}_{control}]\times 100$$

### Brine Shrimp Cytotoxicity Assay

The larvae of *Artemia franciscana* were obtained by decapsulation of sterile cysts as mentioned in Kiran et al. (2016). Briefly, the cysts were aerated in sterile seawater and oxygenated continuously using aerator pumps. After 24 h incubation at room temperature (25–29°C), the freshly hatched nauplii (larvae) were collected by a micropipette. The nauplii were transferred into a 96 deep well plate with 10 nauplii per plate and different concentration of lipopeptide (25–200 μg/ml). The assay was performed in triplicates. Wells were examined under the binocular stereomicroscope (Optica) and the numbers of live and dead (non-motile) nauplii in each well were counted after 24 h.

### Microtitre Plate Assay

The clinical strains of *S. aureus* were collected from Jawaharlal Institute of Postgraduate Medical Education and Research (JIPMER), Puducherry. The antibiogram pattern was tested as per CLSI Guidelines: Clinical and Laboratory Standards Institute (2009) to select multi drug resistant strains. *S. aureus* biofilm was allowed to form on microtitre plate wells containing lipopeptide of varying concentration (25 μg–150 μg/ml). The assay plates were prepared with 10 μl of overnight culture of *S. aureus* diluted upto growth OD of 0.005 and was inoculated into 200 μl of Luria- Bertani broth. The plates were kept under static conditions for 72 h at 37°C. After incubation the planktonic cells were removed by gentle pipetting, and the adhered cells were stained with 0.1% crystal violet and the amount of biofilm formation was quantified at 595 nm using a microplate reader (Labnics) (Kiran et al., 2010a).

### Antibiofilm Effect of Lipopeptide

The antibiofilm activity of lipopeptide was visualized using confocal laser scanning microscopy (CLSM). Effective concentration of lipopeptide 125 μg/ml was added to the Erlenmeyer flask containing LB broth (test) and the flask without lipopeptide was set as the control. The sterile glass slide was immersed into the broth using a sterile forceps. The broth was then inoculated with 1 ml of *S. aureus* overnight culture. After incubation at 37°C for 72 h, the glass slide was washed with sterile distilled water and stained with Baclight kit (Invitrogen) following manufacturer’s instructions. The glass slide was then stained with equal amount of solution A and solution B (3 ml each), incubated for 15 min and observed under CLSM.

### Evaluation of Lipopeptide as a Fat Replacer in Muffin Preparation

In order to evaluate the characteristics of lipopeptide on muffin preparation, the muffins were incorporated with varying concentration of lipopeptide between 0.50 and 1%. The muffin batter formulation was carried out as per Zahn et al. (2010) with necessary modifications. The ingredients used for muffin preparation include 36.57% wheat flour, 11.7% margarine, 22.30% sugar, 17.18% whole egg, 11.7% skim milk, 0.52% baking powder, and 20% water (positive control batter for muffin preparation). In the test, baking powder and egg was replaced with 0.50–1% lipopeptide. The mixture without egg was set as the negative control. The formulated dough was placed in the muffin tray and baked in a preheated oven at 180°C for 20 min until the appearance of light brown color. The prepared muffin was sealed in an air tight container for the textural analysis.

### Textural Evaluation

The textural characteristics of the muffin were analyzed using a Texture analyser (TA–HDplus, Stable Micro Systems, Surrey). The analyser was pre-tested at 1.0 mm/s, test speed at 3.0 mm/s and post-speed at 10.0 mm/s, distance at 10 mm and 12.5 pps data acquisition rate, 50 kg load cell and P75 probe was used to analyze the parameters such as hardness, gumminess, chewiness, springiness, and cohesiveness.

### Color Analysis of Muffin

The color of muffin was analyzed by a Hunter colorimeter (Hunter Lab Associates Inc.) using L^∗^, a^∗^, b^∗^ color space. The L^∗^ represents dark (0)/ light (100), while a^∗^ and b^∗^represents red (+a) to green (+a) and yellow (+b) to blue (-b). The color was analyzed in triplicates and the mean was recorded.

## Results and Discussion

### Isolation, Screening, and Identification of Biosurfactant Producer

**FIGURE 1:**
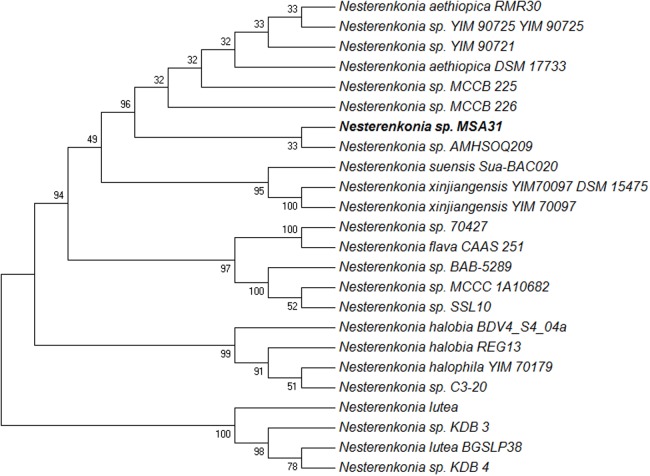
Phylogenetic tree of MSA31 showing representatives of *Nesterenkonia* sp. The evolutionary history was inferred using the Maximum Parsimony method. Tree #1 out of 9 most parsimonious trees (length = 89) is shown. The consistency index is (0.821429), the retention index is (0.938272), and the composite index is 0.780136 (0.770723) for all sites and parsimony-informative sites (in parentheses). The percentage of replicate trees in which the associated taxa clustered together in the bootstrap test (1000 replicates) are shown next to the branches.

A total of 33 morphologically distinct actinomycetes were isolated from the marine sponge *F. cavernosa*. All the isolates were screened for biosurfactant production. The isolate MSA31 was selected based on high emulsification activity (75%) and surface tension reducing property of 34.6 mN/m with critical micelle concentration of 18.6 μg/ml. The isolate MSA31 showed positive results in screening tests which include emulsification, drop collapsing and oil displacement. In drop collapsing test, a flat drop was observed whereas in oil displacement method, a clear diameter of 8 mm corresponding to the area of 64.24 mm^2^ was observed. Emulsification activity, surface tension and the screening tests confirmed the isolate MSA31 as a biosurfactant producer. The enzymes and bioactive molecules produced by the sponge associated bacteria are economically important due to their novelty and remain active even in extremophilic conditions such as elevated salt concentration, wide range of pH, and higher temperatures. The isolate MSA31 grew optimally at increased salt concentration upto 5% and pH 6–10. Similar pH tolerance was reported for *Nesterenkonia alba* which grow optimally at pH 9–10 (Luo et al., 2009). Delgado et al. (2006) reported that *N. aethiopica*, a moderately halophilic strain can grow at salt concentration up to 3% and pH 9.0. There were a very few reports available on sponge associated actinobacteria for lipopeptide production (Gandhimathi et al., 2009; Kiran et al., 2010b). The PCR amplified KS (keto synthase) domain from the sponge associated actinobacteria envisages that the biosynthetic pathway of biosurfactants might have mediated through PKS (polyketide synthase) biosynthetic gene clusters (Selvin et al., 2016). Taxonomic affiliation based on the 16S rRNA sequence of the isolate MSA31 was retrieved from the classifier program of RDPII (Ribosomal Database Project II). The 16S rRNA sequence of the isolate MSA31 was analyzed using a megablast tool of GenBank^3^. Based on the closet matches with *Nesterenkonia* strains, the isolate MSA31 was taxonomically identified as *Nesterenkonia* sp. (**Figure 1**). The closet representative of maximum homologous (98–99%) sequences of each strains was obtained from seqmatch program of RDPII and was used for the construction of phylogenetic tree. The sequence obtained were deposited in genbank with accession number KY969127. A *Nesterenkonia* sp. isolated from Aran Bidgol lake (Iran) was used for the production of halophilic α-amylase with possible application in starch processing industries, baking, brewing, textile, and distillery industries (Shafiei et al., 2012).

### Characterization of Biosurfactant

**FIGURE 2:**
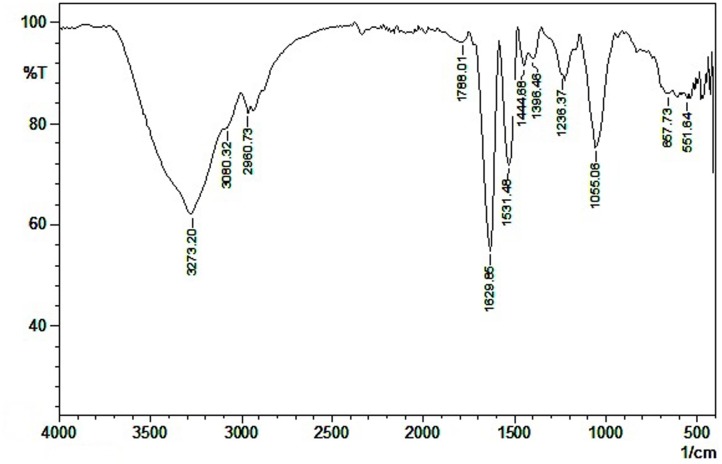
Characterization of biosurfactant produced by MSA31 using FTIR.

Among the various solvents used for extraction, ethyl acetate extract showed highest emulsification activity. The crude biosurfactant was purified in silica gel column chromatography with 80% methanol-water as eluting solvent. The active fractions collected from the column were checked for emulsification activity and analyzed in TLC to determine the purity of the compound. In the TLC plates, a yellow spot corresponding to the Rf value of 0.86 with chloroform: methanol: water (60:30:10) as the solvent system was identified as a biosurfactant (lipid) fraction. The FT-IR spectrum showed a strong broad peak at 3600–3200 cm^-1^ corresponding to the presence of hydrogen bonded –OH or –NH functional groups (**Figure 2**). The carboxylic groups at 1788 cm^-1^ and the sharp peak at 1630 cm^-1^ revealed the presence of amino acid zwitter ion –C = O peak or amide carbonyl peak. The absorbance at 1531 cm^-1^ was attributed to the stretching vibrations of –NH bonds. Similar absorption spectra in FT-IR was reported for lipopeptide in the literature (de Faria et al., 2011; Ghribi and Ellouze-Chaabouni, 2011; Pereira et al., 2013). Weak absorbance signals at 1444 and 1396 cm^-1^ were due to the bending vibrations of C-H bonds associated with alkyl chains. The peak appearing at 1055 cm^-1^ reveals another stretching frequency of C-O functional groups. The FT-IR spectra showed the presence of aliphatic groups combined with peptide moiety, a characteristic feature of lipopeptides. The GC-MS spectra showed the presence of aliphatic chain containing hexyl group.

### ^1^H NMR Analysis

The ^1^H NMR spectra showed broad multiplicity peaks between 8.3 and 7.5 ppm, which typify amino acid amide protons and confirm the presence of several amino acid residues in the molecule (Supplementary Figure S1). The aromatic phenyl group protons appeared as a multiplet in the range of 7.2 to 6.6 ppm and it revealed that the aromatic groups were present in the molecule. Several peaks were observed in the region of 6.6–8.3, which indicates the presence of amino acid amide protons as reported by Pereira et al. (2013). The signals appearing in the region between 5.2 and 4.2 ppm showed the presence of amino acid Hα resonance. The NMR peaks in the region of 5.2 were similar to the peaks reported earlier (Kowall et al., 1998; Liu et al., 2009). The peak at 3.2 ppm to 2.9 ppm revealed the protons attached with the acid functional group. The aliphatic –CH_2_ protons appeared in the range of 2.4 to 1.6 ppm. The singlet peaks appeared at 1.2 and 0.9 ppm confirmed the presence of –CH_3,_ which was attached with quaternary carbon.

### ^13^C NMR Spectra Analysis

In the ^13^C NMR analysis the peak at 173 ppm revealed an –NH_2_ group substituted quaternary carbon in the molecule, it may be due to presence of amino acid group in the molecule (Supplementary Figure S1). The peaks at 155 ppm may be of an electronegative group attached to the aryl group, this reveals that molecule contains the electronegative groups. Peaks in the range of 136, 130, and 117 ppm showed the molecule contains aromatic groups. The peaks in the range of 73, 58, and 53 ppm revealed the presence of aliphatic groups attached to electronegative atoms, trisubstituted or tetrasubstituted carbons. The chemical shift peaks in the range of 32, 30, 25, 20, 16, 12 ppm revealed the presence of several aliphatic groups present in the molecule. The spectral data revealed the compound produced by MSA31 was a lipopeptide moiety containing aromatic aminoacid and also revealed the presence of aliphatic groups.

### Stability of Biosurfactant

**FIGURE 3:**
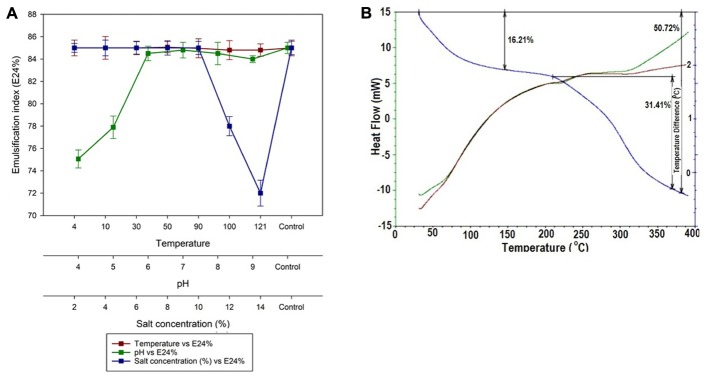
**(A)** Stability of lipopeptide at increased temperature, pH and salt. The temperature stability is shown in **(B)** and the data indicates the compound was highly stable.

Stability of biosurfactant is an important criteria required for its application in food industry. The lipopeptide based biosurfactant produced by MSA31 was found to be thermo-stable in all the temperature ranges tested (**Figure 3A**). Stability was maintained even after autoclaving at 121°C and there was no change in emulsification activity even at low temperature 4°C. This property makes the lipopeptide from MSA31 suitable for ice cream and cosmetic industries. The emulsification activity (85%) of biosurfactant MSA31 was retained at 10% salt concentration and at pH of 6.0–9.0.

In TGA analysis, due to loss of water molecule a change in the mass was observed at 150°C followed by second phase of degradation at 240°C. Maximum degradation occurred at 260°C with weight loss of 50.725%. The DSC data was used to determine the phase transitions of lipopeptide from 0 to 400°C. The DSC thermogram showed exothermic peak of biosurfactant with crystallization temperature (*T*_c_) of 80°C and the melting peaks were found at 240°C and 320°C. The thermal stability of the lipopeptide based on TGA analysis is shown in **Figure 3B**. Lipopeptide produced by MSA31 was found to be thermostable as revealed by DSC analysis. Similar thermostable lipopeptide were produced by *Nocardiopsis* sp*., Brevibacterium aureum*, and *Aneurinibacillus thermoaerophilus* MK01 (Gandhimathi et al., 2009; Kiran et al., 2010b; Sharafi et al., 2014).

### Antioxidant Activity and Toxicity Evaluation

#### DPPH Assay

**FIGURE 4:**
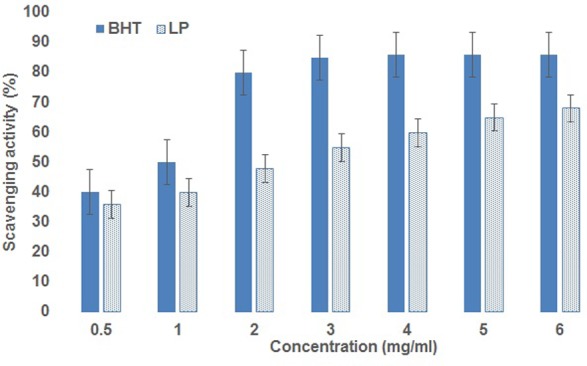
Antioxidant activity of lipopeptide, the activity increases with the concentration of lipopeptide

The DPPH activity of the lipopeptide from MSA31 was compared with the control BHT (**Figure 4**). A concentration dependent antioxidant activity of biosurfactant was recorded and highest activity was obtained at 6 mg/ml. Antioxidant property is an important characteristics of food products as it reduces the risk of coronary heart diseases and is also effective against degenerative diseases. The scavenging activity of lipopeptide MSA31 at 6 mg/ml was 65% which was higher than the scavenging activity recorded for *P. hubeiensis* which showed 50.3% with 10 mg/ml of mannosyl erythritol lipid (Takahashi et al., 2012). The high antioxidant activity of lipopeptide MSA31 may be due to the presence of unsaturated fatty acid present in the molecule. The acute toxicity of the lipopeptide MSA31 was evaluated using the brine shrimp cytotoxicity assay. The assay results revealed the lipopeptide was non-toxic to the brine shrimp *nauplii* in all the concentrations tested (up to 200 μg/ml). Previous reports related to the toxicity of lipopeptide were evaluated in *in vivo* toxicity assays in mice models which concluded that 2,000 mg/kg of body weight dose showed no mortality and normal behavioral indexes (Sahnoun et al., 2014). Park et al. (2006) reported that LD_50_ of surfactin in mice was above 2 g/kg. Biosurfactant produced by *Serratia marcescens* was administered to the mice orally at the dose of 5 g/kg and no toxic effect was noticed in the mice (Anyanwu et al., 2011). The available reports evidenced that lipopeptide and rhamnolipid biosurfactants in general were non-toxic biomolecules which can be used as food additives. According to Sahnoun et al. (2014) the lethal concentration of biosurfactant determined in the animal model was far higher than the threshold concentrations of food additives permitted by Food and Agricultural Organization and World Health Organization. In this study, brine shrimp cytotoxicity and phytotoxicity assays (Supplementary Figure S2) showed the non-toxic nature of lipopeptide biosurfactant.

#### Antibiofilm Effect of Lipopeptide

**FIGURE 5:**
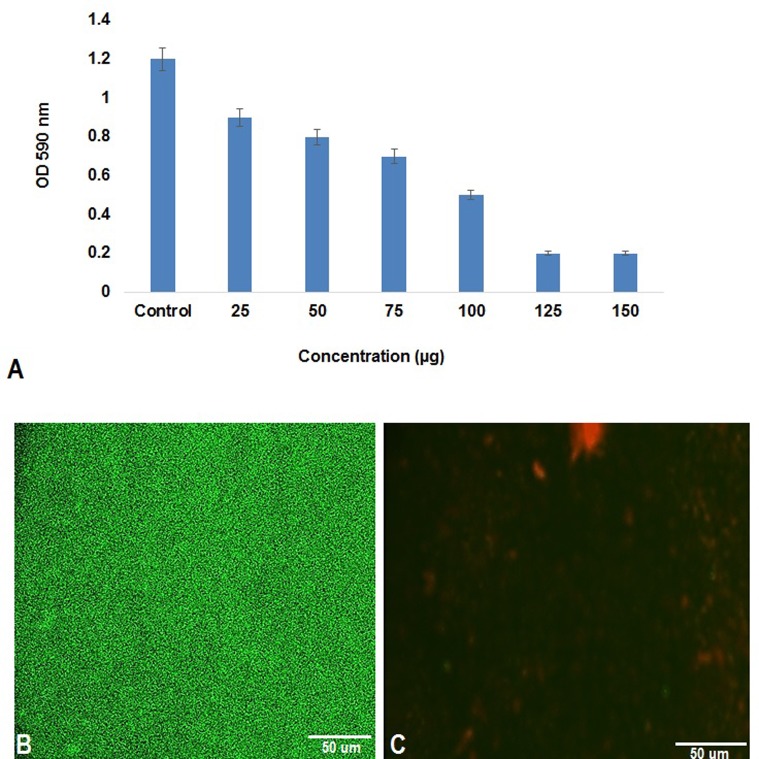
Antibiofilm activity of lipopeptide MSA31 against MDR pathogen *S. aureus*. **(A)** Microtitre assay showing antibiofilm effect of Lipopeptide. **(B)** Is a control biofilm and **(C)** lipopeptide treated biofilm visualized by baclight staining.

Microtiter plate assay showed lipopeptide MSA31 at a concentration of 125 μg/ml effectively reduced 90% of biofilm formed by *S. aureus* when compared to the control. It was observed that antibiofilm activity increases with increase in the concentration of the lipopeptide MSA31 (**Figure 5A**). The confocal microscope images evidenced that the lipopeptide was effective in controlling *S. aureus* biofilm formation (**Figures 5B,C**). The CLSM observation showed that the control biofilm was stained with green indicating live biofilm associated cells of *S. aureus* and the test biofilm was stained red indicating the dead cells of *S. aureus.* Microtiter plate assay and CLSM images showed lipopeptide at a concentration of 125 μg effectively inhibited the biofilm of MDR pathogen *S. aureus.* Reports showed that lipopeptides from *Bacillus* sp. and *Paenibacillus* sp. were known to inhibit/disperse biofilms (Price et al., 2007; Kim et al., 2009; Quinn et al., 2012).

### Effect of Lipopeptide on Muffin Texture

Textural property is an important parameter in determining the quality and sensory characteristics of the muffin. The lipopeptide incorporated muffin showed reduction in hardness, chewiness and gumminess when compared to positive and negative control (**Table 1**). Among the various concentrations of lipopeptide, 0.75% was found to be effective in enhancing the softness and overall quality of the muffin. Muffin prepared using 0.75% lipopeptide had higher level of springiness, when compared to the control. According to Tess et al. (2015), a high springiness value was associated with freshness and high quality of the muffin. Thus 0.75% lipopeptide incorporated muffin was in good quality with higher springiness values. Cohesiveness is defined as the compression energy required for breaking down the food product for swallowing. The muffin prepared with 0.75% lipopeptide showed increased cohesiveness and therefore less compression energy was required to break it down. This property was an indication of soft nature of muffin. Negative control showed higher value of chewiness indicating the hard nature of muffin and 0.75% of lipopeptide incorporated muffin showed very less value of chewiness indicating the soft nature of muffin. Overall, the muffin prepared using 0.75% lipopeptide showed decreased hardness, chewiness and gumminess with increased springiness and cohesiveness.

**Table 1:** Shows texture analysis data of the muffin, the values represented are the mean of triplicate experiments.

TPA parameters	Positive control	Negative control	0.5% LP	0.75% LP	1% LP
Hardness	1978 ± 2.0	2999 ± 3.60	1675 ± 4.0	958 ± 4.35	1345 ± 3.60
Chewiness	1128 ± 3.0	2150 ± 2.64	1115 ± 3.0	485 ± 3.60	900 ± 4.58
Gumminess	1288 ± 2.64	1925 ± 4.58	1188 ± 5.19	515 ± 3.46	855 ± 2.64
Springiness	0.99 ± 0.18	0.81 ± 0.02	0.96 ± 0.12	1.1 ± 0.17	0.98 ± 0.08
Cohesiveness	0.66 ± 0.05	0.61 ± 0.02	0.65 ± 0.05	0.72 ± 0.04	0.67 ± 0.78


*The data is presented as mean ± standard deviation. The data shows that the muffin prepared using lipopeptide exhibited decreased hardness, chewiness and gumminess with increased springiness and cohesiveness values.*

### Color

**FIGURE 6:**
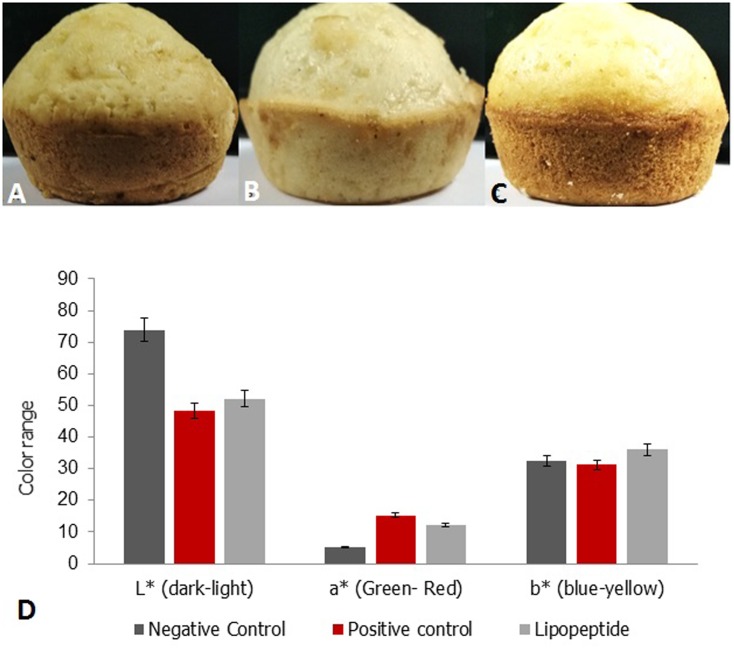
Color analysis of muffin **(A)** Positive control muffin (egg and baking powder), **(B)** Negative control **(C)** muffin prepared using 0.75% of lipopeptide. **(D)** Color analysis of muffin L^∗^ represent the color range of lightness to darkness as per the color scale standards of the HunterLab benchtop color measurement systems (ıColorFlex EZ), a^∗^ represents green (–) to red (+), b^∗^ represents blue (–) to yellow (+). The addition of lipopeptide showed improved original color pattern of muffin to yellow color.

Color is an important factor to increase the appeal of the food products and is directly related to acceptance and taste (Chakraborty et al., 2015). The L^∗^, a^∗^, and b^∗^ values of prepared muffins are given in **Figure 6**. The muffin prepared by using lipopeptide showed slight increase in the b^∗^ value which indicates the yellow colouration. The b^∗^ value of lipopeptide muffin was found to be 36 when compared to the negative and positive controls of 32.37 and 34.2, respectively. The L^∗^ value was higher in the negative control (without egg and baking powder), which indicates light color of the muffin and a^∗^ value was found to be almost similar to the positive control and lipopeptide incorporated muffin. Normally in baked food products, changes in the natural color of the flour during baking is not uncommon due to Maillard reactions. The muffin prepared using lipopeptide MSA31 was light yellow in color as evident from b^∗^ value and the appeal was almost similar to the positive control. When Jambolan was incorporated into the muffin, the anthocyanin pigments in the Jambolan imparted dark color to the muffin (Singh et al., 2015).

## Conclusion

In this study, actinobacterial strain *Nesterenkonia* sp. MSA31 was isolated from a marine sponge *F. cavernosa*. The strain MSA31 was found to be the highest biosurfactant producer among the 33 isolates screened. Based on the stability index, the lipopeptide MSA31 was characterized as “halo-alkali and thermal tolerant biosurfactant” which can be used as emulsifier and emulsion stabilizing agent in the food industry. The lipopeptide MSA31 was non-toxic, showed antibiofilm activity against a prominent food pathogen *S. aureus.* The emulsification property with antioxidant activity showed that the lipopeptide MSA31 can be highly beneficial to the food industry. Colors is an important factor to increase the appeal for the food products. The muffin prepared using lipopeptide showed light yellow in color, and the appeal was almost similar to the positive control. This study provided a new insight for the food industry as incorporation of lipopeptide emulsifier could improve the quality of food products with antioxidant activity as well as antibiofilm activity against *S. aureus*.

## Author Contributions

AS, NP, SP, and GBP performed laboratory experiments and data analysis, GSK written the manuscript and JS designed and guided the work.

## Conflict of Interest Statement

The authors declare that the research was conducted in the absence of any commercial or financial relationships that could be construed as a potential conflict of interest.

## Footnotes

1 www.ebi.ac.uk/cgi-bin/clustalw/

2 www.megasoftware.net

3 http://www.ncbi.nlm.nih.gov/
