# Supplementary material for: Production of Lipopeptide Biosurfactant by a Marine Nesterenkonia sp. and Its Application in Food Industry
Source: Front Microbiol. 2017 Jun 28;8:1138. doi: 10.3389/fmicb.2017.01138 (PMC5488535; doi:10.3389/fmicb.2017.01138)
Supplement: Supplementary file 1 [file Presentation_1.pdf]

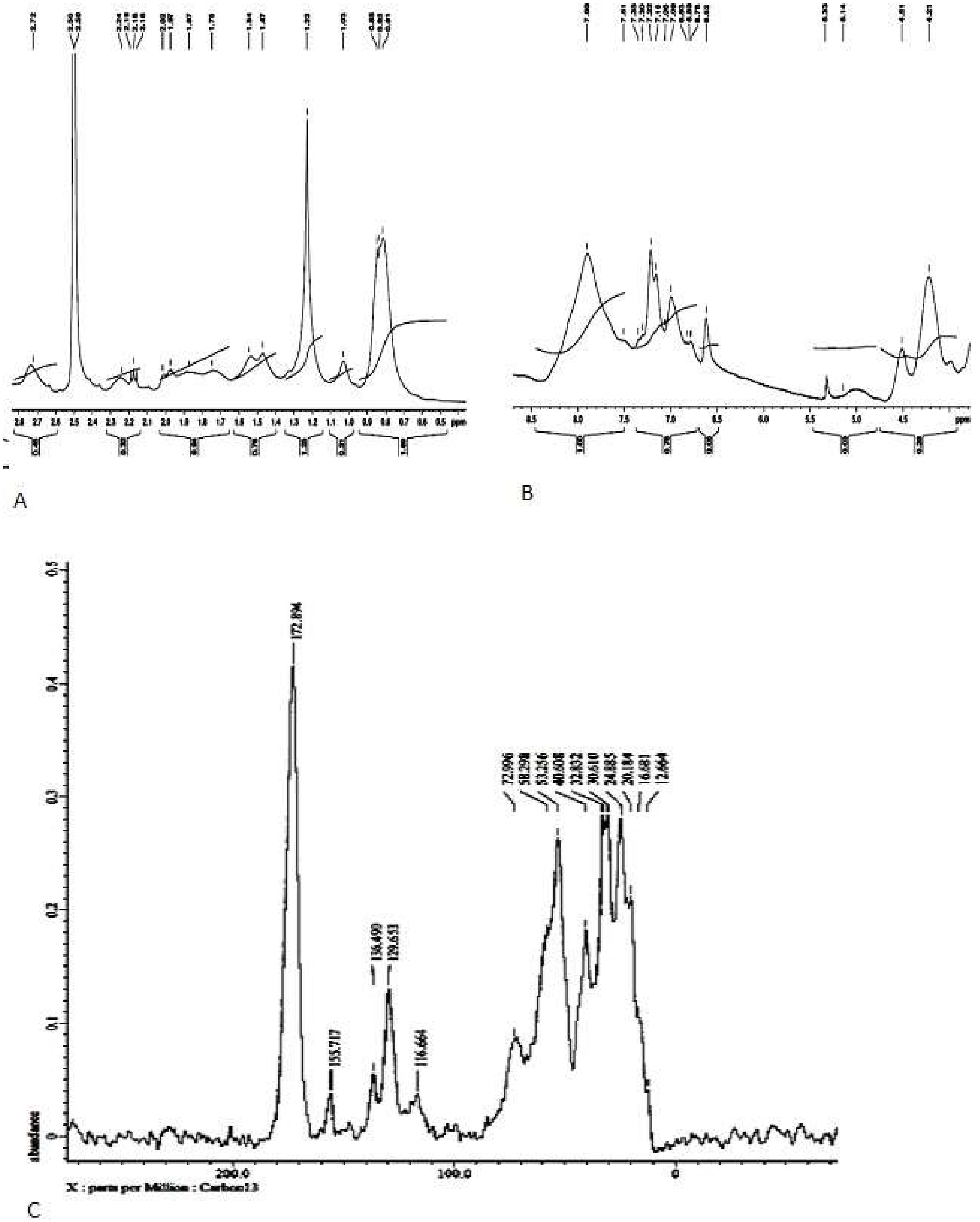

Supplementary Figure S1. H-NMR (A-B) and C-NMR (C). The peaks obtained using the spectroscopy revealed the presence of aromatic amino acids and fatty acid molecule.

Seed Germination Assay

Phytotoxic effect of the lipopeptide was analysed using seed germination assay (Ruffino et al, 2007). Different concentration of biosurfactant ranging from 100-200 (mg/ml) was analysed on the seeds of *Vigna radiate*. A total of 12 Seeds were taken and placed in two petridish containing lipopeptide. The seeds were dipped in the solution of lipopeptide for 10min and the petriplate with water serve as the control. The soil was then added to the paper cups and four seeds were placed in the respective cups and incubated at 30°C for 4days. All the assays were performed in duplicate. After 4 days of growth, the plant was observed for the growth with number of leaves, primary and secondary root development.

Result

The germination of seeds was well noticed from the third day of the experiment. When compared to the control, the number of seed germinated and the growth was excellent in the soil supplemented with lipopeptide. When the seeds were exposed to lipopeptide solution the plant was healthy with healthy root formation when compared to the control. This proves the lipopeptide produced by MSA31 was non-toxic and it improved the plant with primary and secondary root formation.

Reference

Rufino, R.D., Sarubbo, L.A., and Campos-Takaki, G.M. (2007). Enhancement of stability of biosurfactant produced by *Candida lipolytica* using industrial residue as substrate. *World Journal of Microbiology and Biotechnology*, 23 (5) 729-734.
